# Ruptured Right Broad Ligament Ectopic Pregnancy in a Patient With Prior Right Salpingo-Oophorectomy: A Case Report

**DOI:** 10.7759/cureus.8276

**Published:** 2020-05-25

**Authors:** Erum Azhar, Landen Green, Salma Mohammadi, Abdul Waheed

**Affiliations:** 1 Obstetrics and Gynecology, Maimonides Medical Center, Brooklyn, USA; 2 Public Health Sciences, Penn State University College of Medicine, Hershey, USA; 3 Family Medicine, Wellspan Good Samaritan Hospital, Lebanon, USA; 4 Family and Community Medicine, Penn State University College of Medicine, Milton S. Hershey Medical Center, Hershey, USA

**Keywords:** ruptured broad ligament ectopic pregnancy, abdominal pregnancy, interligamentous pregnancy

## Abstract

Ectopic pregnancy can be a life-threatening cause of acute abdomen. Broad ligament pregnancy accounts for 1% of ectopic abdominal pregnancies and complications can be calamitous. This case report highlights a 27-year-old G2P0010 female who presented with amenorrhea and acute right lower quadrant and pelvic pain. By last menstrual period (LMP), she was at seven weeks and two days gestation. Her past surgical history was significant for a prior right salpingo-oophorectomy. The physical examination was significant for severe right lower quadrant tenderness with guarding. The urine pregnancy test was positive with the serum quantitative beta-human chorionic gonadotrophin (Beta hCG) of 28011 MIU/ML (normal range <5 MIU/ML). The transvaginal ultrasonography demonstrated an empty uterus and a gestational sac containing a fetal pole in the right adnexal area. The crown-rump length was 7.2 mm, consistent with six weeks and four days, with a positive fetal heart rate and moderate free fluid in the cul-de-sac. The patient was taken for immediate diagnostic laparoscopy, which was converted to open laparotomy due to active bleeding from the right broad ligament and pelvic wall close to large pelvic vessels. In addition to the hemoperitoneum, intraoperative findings revealed a normal left fallopian tube and ovary and absent right fallopian tube and ovary. Right ureterolysis was done and hemostasis of the bleeding broad ligament and right pelvic sidewall was established. An adherent tissue was dissected from the right broad ligament and sent to pathology. The patient did well postoperatively. The final pathology showed an ectopic pregnancy with immature chorionic villi in the broad ligament. The diagnosis of ectopic pregnancy in the broad ligament is challenging. The location could be close to the major pelvic vessels and anatomic structures like the ureter and bowel, hence, it can cause massive hemorrhage with maternal morbidity and mortality. Diagnosis is often missed preoperatively and made intraoperatively. Hence, we emphasize that this differential be considered in reproductive-aged women who present with atypical presentations of acute abdomen and amenorrhea.

## Introduction

Ectopic pregnancy is defined as the implantation of the fertilized egg outside the endometrial cavity of the uterus. It occurs in 2% of all pregnancies, with 95% of the cases occurring in the fallopian tube [1]. Abdominal pregnancy is a rare type of ectopic pregnancy, with a reported incidence of 1:10,000 to 1:30,000 pregnancies [2]. It could be life-threatening with maternal mortality eight times greater than the usual tubal ectopic pregnancies [3-4]. Fortunately, abdominal ectopic pregnancies account for only approximately 1% of the ectopic pregnancies [2-3].

Abdominal pregnancy is reported in the literature at different anatomic sites, including the broad ligament, the pouch of Douglas, pelvic sidewall, spleen, bowel, omentum, and at different gestational ages [2,5-7]. The location of the abdominal pregnancy could be close to the major pelvic vessels and anatomic structures, like the ureter and bowel, hence, can cause massive hemorrhage and maternal morbidity.

Diagnosis is often missed preoperatively and made intraoperatively, sometimes at a later gestational age, which could result in significant hemorrhage and organ injury [6,8]. Abdominal pregnancy having live birth has been reported by many authors [9-12]. However, Martin et al. reported fetal death in 15 cases of advanced abdominal pregnancy where expectant management was attempted to get good neonatal outcomes. It is, therefore, important to diagnose and manage abdominal pregnancy early, as the maternal mortality rate could be as high as 20% and the perinatal mortality rate is between 40%-95% [13].

## Case presentation

A 27-year-old woman, gravida 2, para 0-0-1-0, presented to the emergency department with severe right lower quadrant pain and amenorrhea. By her last menstrual period, she was seven weeks and two days pregnant. The patient reported that the pain started three days ago but became severe, which prompted her to come to the emergency department. She denied any vaginal bleeding or passage of tissue. Her obstetrical history demonstrated one prior incomplete abortion at seven weeks gestation one and a half years prior, necessitating a dilatation and curettage. Her gynecological history was significant for a prior laparoscopic salpingo-oophorectomy one-year ago at another hospital for torsion of right adnexal mass that revealed endometriotic ovarian cyst and paratubal cyst of the fallopian tube on pathology. Since her last surgery, she had not been using any form of contraception. Apart from this, all other past medical, surgical, and family history was unremarkable. Her vital signs were significant with a blood pressure of 118/73, a heart rate of 102, and an oxygen saturation of 99% on room air. Physical examination of the abdomen revealed right lower quadrant tenderness on deep palpation with guarding and rebound. Pelvic examination revealed tenderness in the right pelvic area with no adnexal masses felt and a closed cervical os.

**Figure 1:**
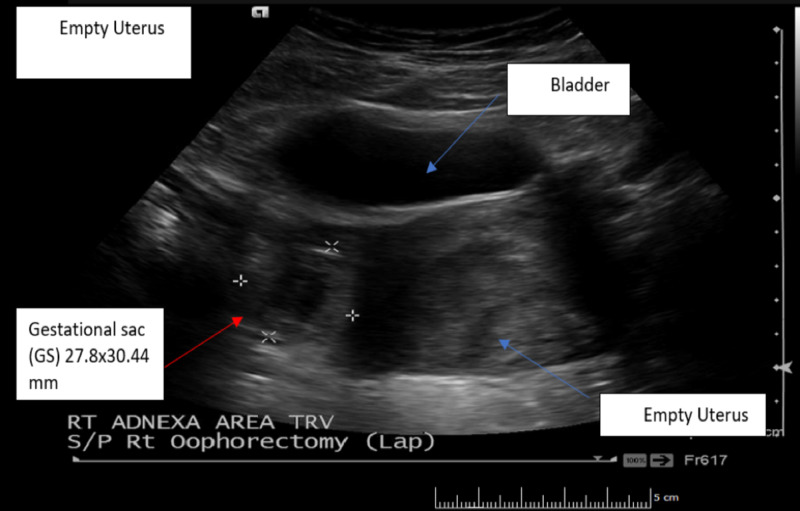
Pelvic ultrasound (transverse view): showing full bladder, empty uterus with endometrial lining thickness of 17 mm, and a gestational sac (GS) in the right adnexa measuring 27.8x30.44 mm

**Figure 2:**
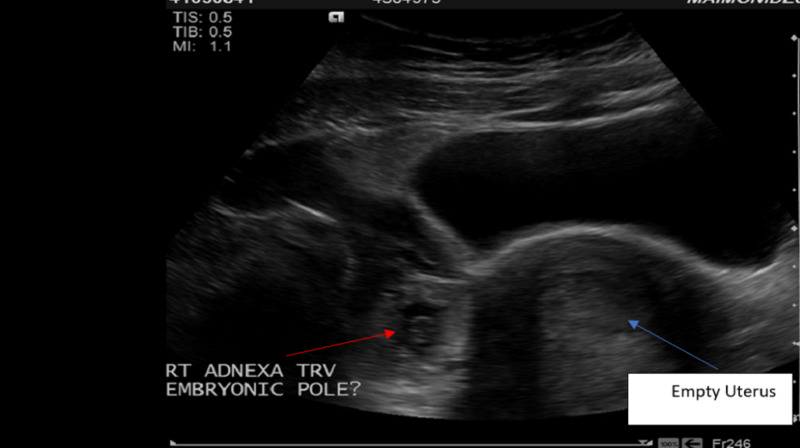
Pelvic ultrasound (transverse view): embryonic pole in the gestational sac in the right adnexal area

**Figure 3:**
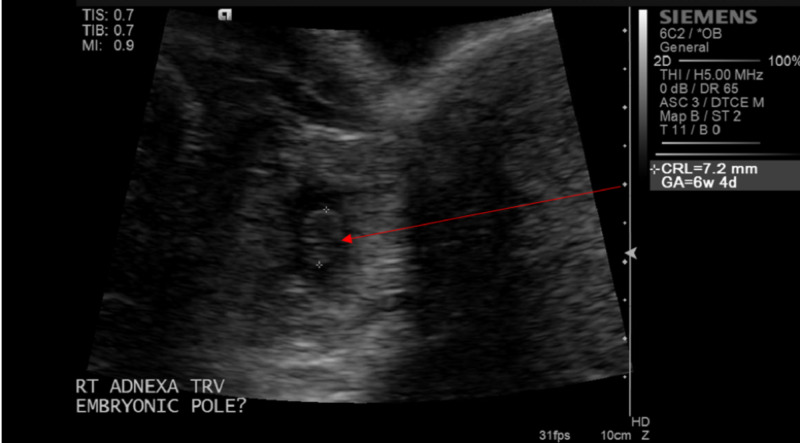
Pelvic ultrasound (transverse view): ectopic pregnancy with a crown-rump length (CRL) of 7.2 mm (gestational age six weeks and four days) in the right adnexal area

**Figure 4:**
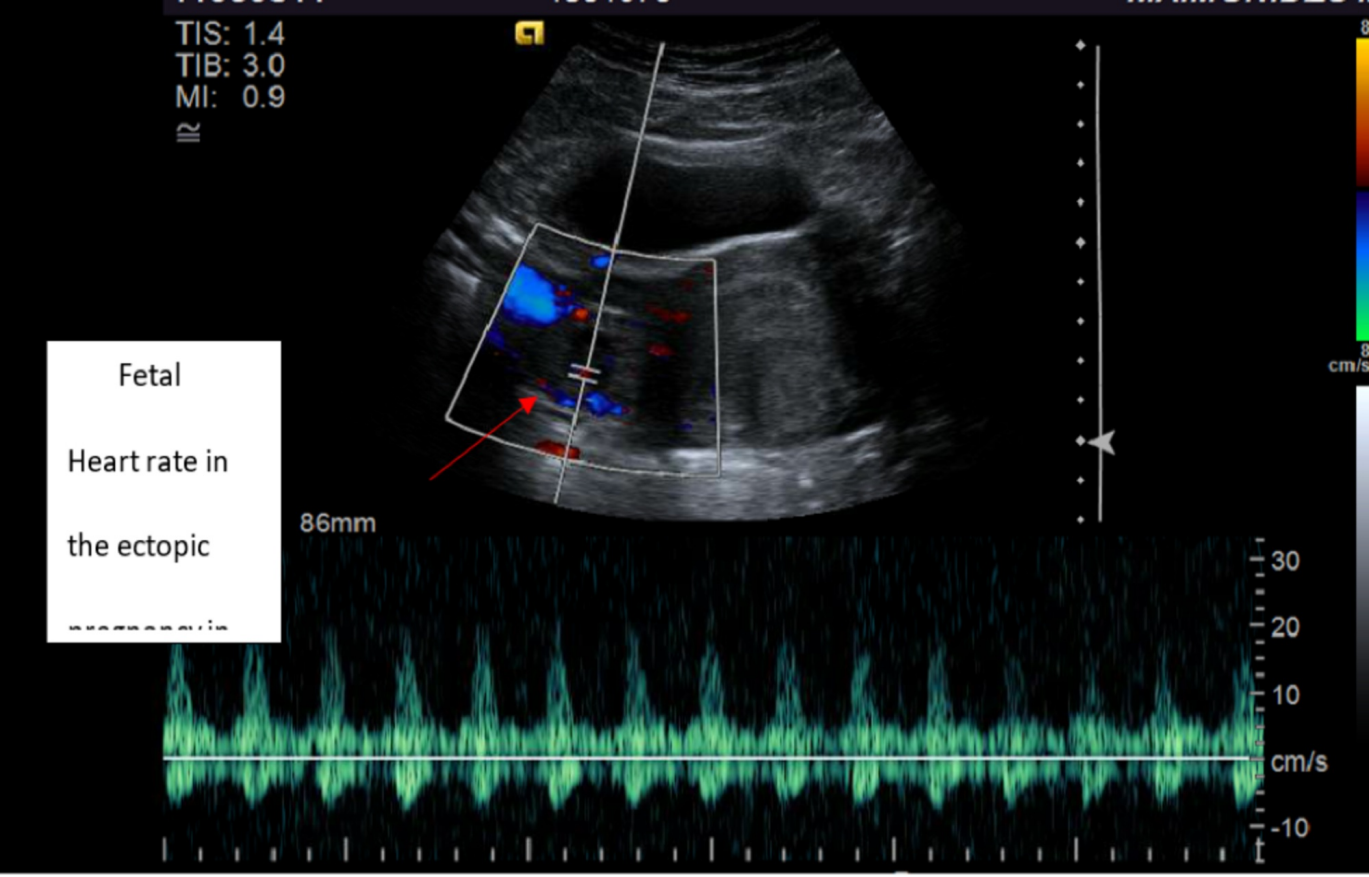
Pelvic ultrasound (transverse view): fetal heart rate demonstrated in the ectopic pregnancy in the right adnexal area

The urine pregnancy test was positive with the serum quantitative beta-hCG of 28011 MIU/ML (normal range <5 MIU/ML). Transvaginal ultrasonography demonstrated an empty uterus with an endometrial lining of 17 mm (Figure 1) and a gestational sac containing a fetal pole in the right adnexal area with a crown-rump length of 7.2 mm consistent with six weeks and four days (Figures 2-3). A positive fetal heart rate was demonstrated (Figure 4) and moderate free fluid was seen in the pelvis. The left adnexa was not visualized. The blood count revealed hematocrit 34% (normal range 37%-47%), white blood count 9.6K/UL (normal range 4.8-10.8 K/UL), and platelet count of 144 (normal range 150-500 K/UL).

Differentials included gynecological and non-gynecological causes of acute abdominal and pelvic pain. Gynecological causes included, but were not limited to, ovarian torsion, ruptured corpus luteal cyst, ruptured ectopic pregnancy, degenerating fibroids, tube-ovarian abscess, endometrioma, and ovarian tumors, both benign and malignant. The non-gynecological causes included in the differentials were ruptured appendix, diverticulitis, colitis, renal colic, pyelonephritis, bowel obstruction, and incarcerated inguinal hernia.

**Figure 5:**
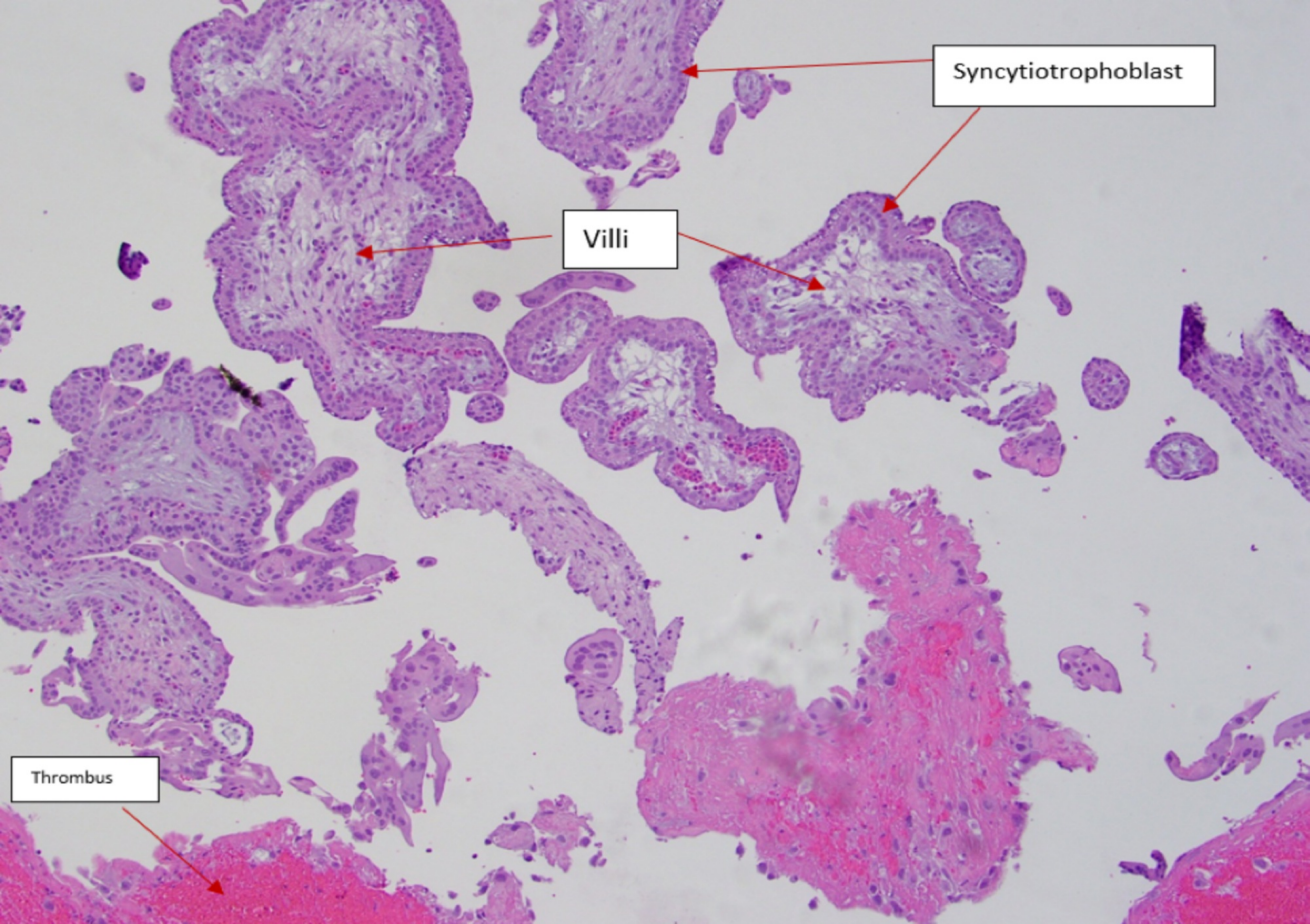
Pathology: rare immature chorionic villi with blood clots and thrombus consistent with a ruptured ectopic pregnancy

The patient’s clinical and ultrasonography findings, along with her prior history of right salpingo-oophorectomy, raised high suspicion for ruptured broad ligament ectopic pregnancy. The patient underwent immediate diagnostic laparoscopy, which revealed a normal left fallopian tube and ovary and an absent right fallopian tube and ovary consistent with her prior history of right salpingo-oophorectomy with hemoperitoneum in the cul-de-sac. There was an active bleeding site with tissue adherent to the right broad ligament and pelvic wall with vascular implants to the pelvic vessels. Hence, the decision was made to convert to an open laparotomy. Right ureterolysis was done and hemostasis of the bleeding broad ligament and right pelvic sidewall was established. The adherent tissue was dissected from the right broad ligament with vascular implants to the pelvic vessels and sent to pathology. The patient received two units of packed red blood cells (pRBCs) intraoperatively with estimated blood loss (EBL) of 2000 ml. The patient did well postoperatively and was discharged home on the fourth postoperative day. Gross pathology demonstrated multiple pieces of brownish-red soft tissue, along with a blood clot measuring 3.5 x 3.2 x 1.2 cm in aggregate. Microscopic evaluation revealed rare immature chorionic villi in the broad ligament and no fallopian tube tissue (Figure 5). She was followed postoperatively at two weeks and six weeks as well and received Depo-Provera for contraception.

## Discussion

Broad ligament pregnancy is also called interligamentous pregnancy. It is a rare, life-threatening form of ectopic abdominal pregnancy and accounts for approximately 1% of all the ectopic pregnancies [2-3]. Most cases of broad ligament ectopic pregnancies reported in the literature are in the first or early second trimester; however, the term broad ligament pregnancy case report has been reported by Deneke [14-17].

The clinical presentation of an abdominal pregnancy depends on its location. It can present similar to a fallopian tube ectopic pregnancy with amenorrhea and pelvic pain. However, if the implantation is close to large pelvic vessels, it can present with massive internal hemorrhage and shock [16]. A pelvic ultrasound can assist with the diagnosis of ectopic pregnancy with the characteristics of an empty uterine cavity and a gestational sac in the surrounding adnexal area. However, it can go undiagnosed until an advanced gestational age, which can complicate the management [5,18]

Abdominal pregnancy is reported in the literature to result from the penetration of the trophoblastic tissue of a tubal pregnancy through the serosa of the fallopian tube and into the mesosalpinx, getting implanted between the leaves of the broad ligament secondarily [18]. Broad ligament pregnancy can also develop if there is a fistula formation for various reasons between the uterine endometrial cavity and the retroperitoneal space between the leaves of the broad ligament. Weakness and separation in a prior surgical cesarean scar or a uterine perforation after an elective or therapeutic abortion can also result in secondary broad ligament implantation [9,18]. Our patient did have a history of a prior dilatation and curettage for incomplete abortion at seven weeks gestation as well as a prior right salpingo-oophorectomy for an endometriotic lesion in the past.

As minimally invasive gynecology surgery has advanced, laparoscopic management of broad ligament ectopic pregnancy has been reported in the literature, especially if the patient is stable or ectopic is unruptured [6,19]. Cho YK et al. reported a case of a multiparous woman with a prior history of bilateral tubal ligation who was diagnosed with right broad ligament ectopic pregnancy [19]. The patient underwent laparoscopic management with the removal of the broad ligament ectopic. Partial cornual resection of the uterine serosa was done, the patient also underwent oophorectomy with bilateral salpingectomy as the vascular implants were extending in the infundibulopelvic ligament. Cosentino et al. also reported a case of a 12 weeks gestation broad ligament ectopic pregnancy, which was managed laparoscopically [6]. The patient underwent left adnexectomy after transperitoneal identification of the left ureteral path and removal of left broad ligament pregnancy with the removal of the ectopic placenta.

Patients with broad ligament pregnancies should be counseled extensively preoperatively regarding the increased morbidity associated with the proximity to the pelvic sidewall and pelvic anatomic structures. The ureters traverse the broad ligament and there might be a need for the repair or removal of the organs damaged by the vascular invasion. Laparoscopic or robotic surgery should be utilized as long as the patient is vitally stable and it is anatomically feasible [15].

Our patient was appropriately diagnosed with broad ligament ectopic pregnancy given her prior history of right salpingo-oophorectomy. Extensive counseling regarding the involvement of the pelvic sidewall, ureters, and large pelvic vessels was done and the patient was explained the possibility of exploratory laparotomy to control bleeding or need for any further organ repair. We started the case laparoscopically, however, intraoperative findings suggested a vascular invasion of placental tissue close to the pelvic vessels on the right with the active bleeding site and hemorrhage. Thus, the decision was made to convert to exploratory laparotomy. The pelvic side walls were dissected, right ureterolysis was done, and placental tissue with vascular implants was removed in its entirety without any complications to the adjacent organs.

## Conclusions

Ectopic pregnancy in the broad ligament is a rare, life-threatening form of abdominal pregnancy with high maternal morbidity and mortality. The rarity of this type of ectopic pregnancy can often make early preoperative diagnosis difficult, hence, it is diagnosed intraoperatively and often at a later gestational age. Thus, we emphasize that this differential diagnosis be considered in reproductive-age women who present with atypical presentations of ectopic pregnancy for the early diagnosis and prevention of the calamitous consequences of morbidity and mortality.
